# Supplementary material for: Metagenome-scale analysis yields insights into the structure and function of microbial communities in a copper bioleaching heap
Source: BMC Genet. 2016 Jan 19;17:21. doi: 10.1186/s12863-016-0330-4 (PMC4717592; doi:10.1186/s12863-016-0330-4)
Supplement: Additional file 1: — Summary of metagenome dataset of samples from the surface-layer mine tailings. (DOC 40 kb) [file 12863_2016_330_MOESM1_ESM.doc]

**Additional file 1** Summary of metagenome dataset of samples from the surface-layer mine tailings.

| **Properties** | **Values** |
| --- | --- |
| Raw sequence reads | 3,349,899 |
| High-quality sequences | 2,941,297 (87.80%) |
| Assembly sequences | 481,688 |
| Total bases | 301,907,459 |
| Max sequence length (bp) | 49,868 |
| Min sequence length (bp) | 301 |
| Mean length (bp) | 626 |
| Mean GC (%) | 60.00 |
| N50 (bp) | 641 |
| Protein-coding sequences | 660,572 |
| CDSs with NCB-nr hits | 535,887 (81.12%) |
| CDSs with COG hits | 517,948 (78.41%) |
| CDSs with COGs | 497,601 (75.33%) |
| CDSs with KEGG hits | 494,721 (74.89%) |
| CDSs with KEGG Orthology | 261,595 (39.60%) |
