# Supplementary figures and images for: Metagenome-scale analysis yields insights into the structure and function of microbial communities in a copper bioleaching heap
Source: BMC Genet. 2016 Jan 19;17:21. doi: 10.1186/s12863-016-0330-4 (PMC4717592; doi:10.1186/s12863-016-0330-4)

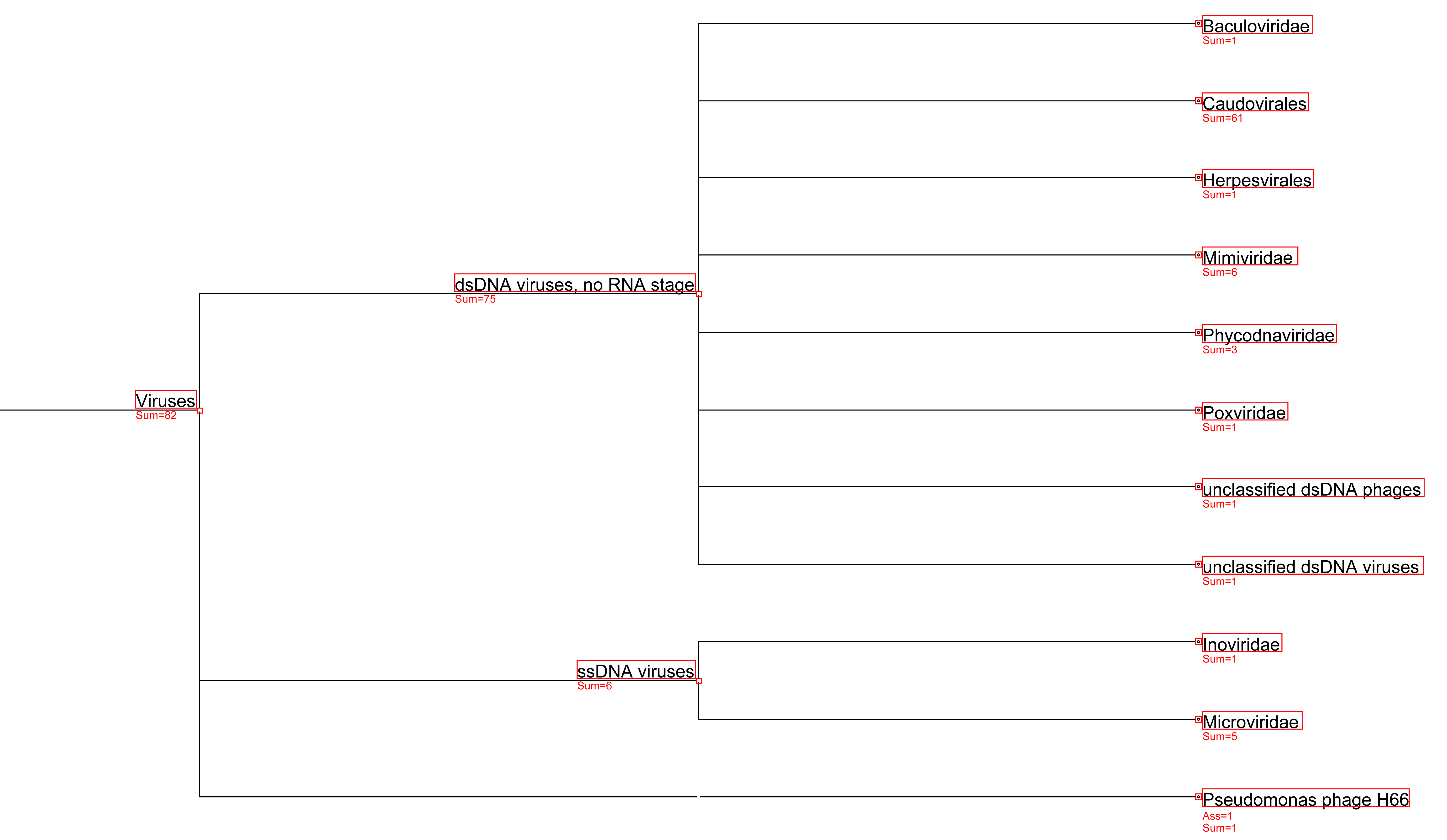

Supplement: Additional file 3: — The viral composition in the metagenome collected from bioleaching heap. (TIF 423 kb) [file 12863_2016_330_MOESM3_ESM.tif]

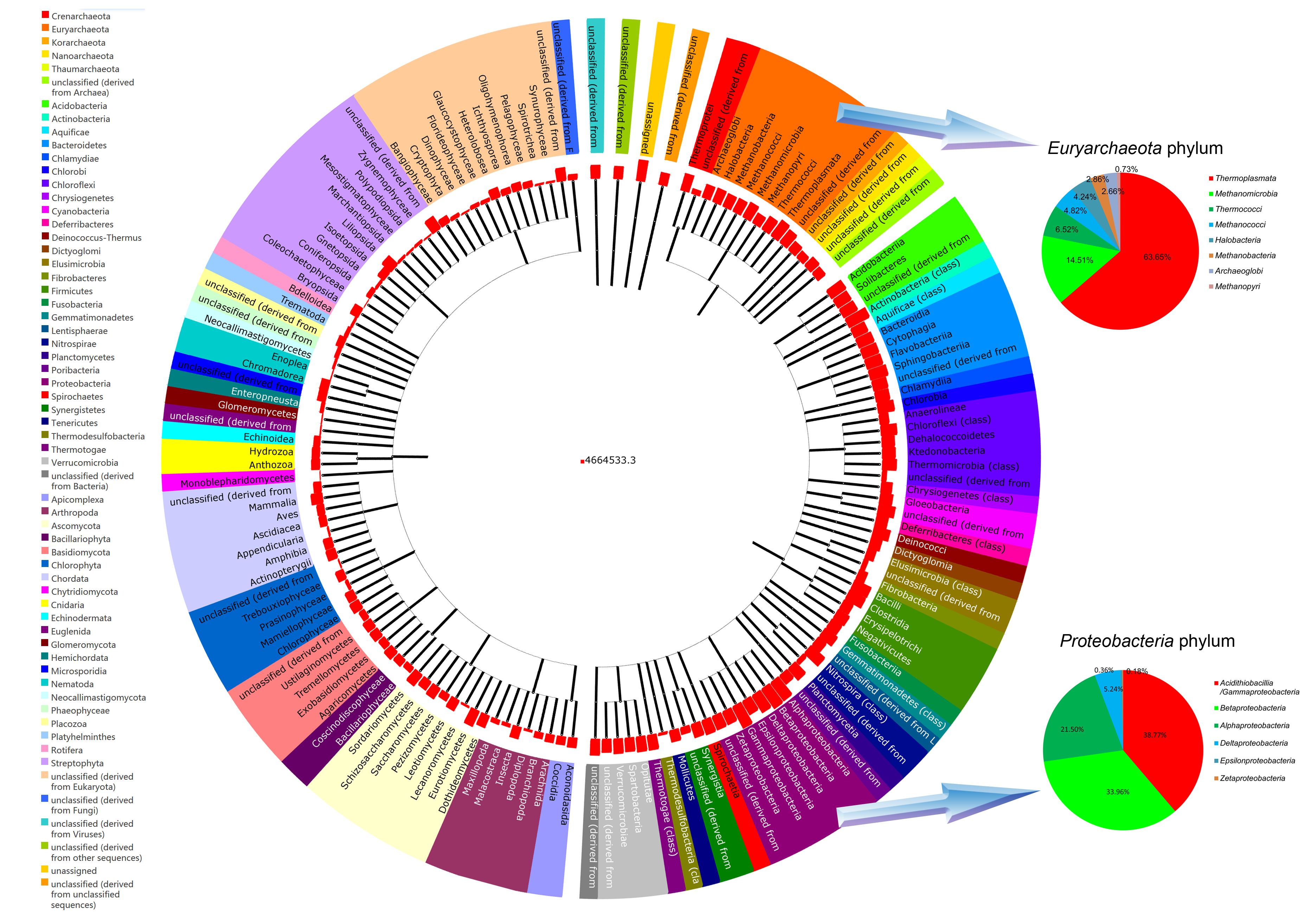

Supplement: Additional file 4: — Phylogenetic tree at the phylum level on the basis of metagenome dataset. The data was compared to M5NR using a maximum e-value of 1e −5, a minimum identity of 60 %, and a minimum alignment length of 15 measured in aa for protein and bp for RNA databases. In addition, display leaf weights as stacked bar, maximum level is class, color by phylum. (TIF 3380 kb) [file 12863_2016_330_MOESM4_ESM.tif]
