# Supplementary material for: Metagenome-scale analysis yields insights into the structure and function of microbial communities in a copper bioleaching heap
Source: BMC Genet. 2016 Jan 19;17:21. doi: 10.1186/s12863-016-0330-4 (PMC4717592; doi:10.1186/s12863-016-0330-4)
Supplement: Additional file 6: — Key genes encoding enzymes related to heavy metal resistance and low pH tolerance. (DOCX 31 kb) [file 12863_2016_330_MOESM6_ESM.docx]

**Additional file 6** Key genes encoding enzymes related to heavy metal resistance and low pH tolerance.

| Gene | Description | No. of CDSs | Most speciﬁc taxon assignment | COG ID | COG category |
| --- | --- | --- | --- | --- | --- |
| Heavy metal resistance | | | | | |
| Arsenate resistance | | | | | |
| *arsC* | Arsenate reductase | 50 | *Dyella*, *Acidithiobacillus* | COG1393 | [P]* |
| *arsR* | Arsenate repressor | 530 | *Acidithiobacillus* | COG0640 | [K] |
| *arsB* | Arsenate membrane efflux pump | 85 | *Acidithiobacillus* | COG1055 | [P] |
| *arsH* | Hypothetical protein | 18 | *Dyella*, *Acidithiobacillus* | COG0431 | [R] |
| Mercury resistance | | | | | |
| *merR* | MerR family transcriptional regulator | 525 | *Acidithiobacillus* | COG0789 | [K] |
| *merA* | Mercuric reductase MerA | 266 | *Thiobacillus*, *Leptospirillum* | COG1249 | [C] |
| *merP* | Periplasmic transport protein MerP | 37 | *Thiomonas*, *Thiobacillus* | NOG79562 | [P] |
| *merT* | Mercuric ion transporter MerT | 46 | *Thiomonas*, *Thiobacillus* | NOG11562 | [P] |
| *merC* | Mercury transport protein MerC | 26 | *Thiomonas*, *Acidithiobacillus* | NOG56224 | [P] |
| *merD* | Mercury co-repressor MerD | 3 | *Acidithiobacillus* | NOG40540 | [K} |
| Cobalt-zinc-cadmium resistance protein | | | | | |
| *czcA* | Heavy metal efflux pump, CzcA family | 676 | *Acidithiobacillus*, *Leptospirillum* | COG3696 | [P] |
| *czcB* | Heavy metal RND efflux membrane fusion protein, CzcB family | 31 | *Thiomonas*, *Leptospirillum* | NOG01644 | [P] |
| *czcC* | Heavy metal RND efflux outer membrane protein, CzcC family | 24 | *Thiobacillus* | NOG19426 | [M] |
| Copper resistance | | | | | |
| Copper ATPase | | | | | |
| *copA* | Copper-translocating P-type ATPase CopA | 134 | *Thiomonas*, *Thiobacillus* | COG2132 | [Q] |
| *copB* | Copper-translocating P-type ATPase CopB | 83 | *Thiomonas*, *Rhodanobacter* | COG3667 | [P] |
| Cus system | | | | | |
| *cusA* | Heavy metal efflux RND transporter CusA | 30 | *Acidobacterium* | COG3696 | [P] |
| *cusB* | Heavy metal efﬂux transporter, MFP subunit (CusB) | 10 | *Acidobacterium* | NOG01644 | [P] |
| *cusC* | Outer membrane heavy metal efﬂux protein (CusC) | 30 | *Acidobacterium* | NOG19426 | [M] |
| Low pH adaption | | | | | |
| Potassium-transporting ATPase | | | | | |
| *kdpA* | Potassium-transporting ATPase subunit A | 249 | *Acidithiobacillus* | COG2060 | [P] |
| *kdpB* | Potassium-transporting ATPase subunit B | 298 | *Acidithiobacillus* | COG2216 | [P] |
| *kdpC* | Potassium-transporting ATPase subunit C | 117 | *Acidithiobacillus* | COG2156 | [P] |
|  | Plasma-membrane proton-efflux P-type ATPase | 106 | *Acidithiobacillus* | COG0474 | [P] |
|  | Voltage-gated potassium channel | 34 | *Acidithiobacillus*, *Leptospirillum* | COG1226 | [P] |
|  | Voltage-gated potassium channel beta subunit | 19 | *Thiomonas* | COG0667 | [C] |
| Monovalent cation/proton antiporters (CPA) | | | | | |
|  | Na^+^/H^+^ antiporter, CPA1 family | 174 | *Acidithiobacillus* | COG3263/ COG0025 | [P] |
|  | Na^+^/H^+^ antiporter, CPA2 family | 301 | *Acidithiobacillus* | COG0475/ COG1226 | [P] |
|  | Na^+^/H^+^ antiporter, NhaA family | 105 | *Acidithiobacillus* | COG3004 | [P] |
| Organic solvents tolerance | | | | | |
| ABC transport system involved in toluene resistance | | | | | |
|  | ABC transporter ATP-binding protein | 255 | *Dyella*, *Rhodanobacter* | COG1131 | [V] |
|  | Toluene tolerance protein Ttg2D | 54 | *Acidithiobacillus* | COG2854 | [Q] |
|  | Mce-related protein (substrate-binding protein) | 32 | *Leptospirillum* | NOG02063 | [R] |
|  | Toluene tolerance protein Ttg2B (permease protein) | 11 | *Thiomonas* | COG0767 | [Q] |
|  | Toluene tolerance protein Ttg2A (ATP-binding protein) | 11 | *Thiomonas* | COG1127 | [Q] |
|  | Aromatic hydrocarbon degradation membrane protein | 84 | *Acidithiobacillus* | COG2067 | [M] |
|  | Organic solvent tolerance protein | 179 | *Thiobacillus*, *Acidithiobacillus* | COG1452 | [M] |
| AcrAB-TolC | | | | | |
| *acrB* | Transporter AcrB/AcrD/AcrF family protein | 148 | *Thiomonas*, *Leptospirillum* | COG0841 | [P] |
| *acrA* | RND family efflux transporter MFP subunit | 225 | *Acidithiobacillus* | COG0845 | [M] |
| *tolC* | TolC family type I secretion outer membrane protein | 158 | *Acidithiobacillus* | COG1538 | [M] |
| Detoxification of hydroxyl radicals | | | | | |
| Peroxidoxin | | | | | |
| *LcPxn1* | Peroxidoxin 1 (AAG40074.1) | 23 | *Leptospirillum* | COG1225 | [O] |
| *LcPxn3* | Peroxidoxin 3 (AAK69587.1) | 109 | *Leptospirillum* | COG0450 | [O] |
| *LdPxn1* | Peroxidoxin 1 (AAK82654.1) | 30 | *Thiobacillus*, *Leptospirillum* | COG0450/COG1225 | [O] |
|  | Peroxidase/catalase | 192 | *Thermoplasmatales* group | COG0376 | [P] |

*COG category: [P] Inorganic ion transport and metabolism; [K] Transcription; [R] General function prediction only; [C] Energy production and conversion; [M] Cell envelope biogenesis, outer membrane; [Q] Secondary metabolites biosynthesis, transport and catabolism; [V] Defense mechanisms; [O] Posttranslational modification, protein turnover, chaperones.
